# Another Appeal

**Published:** 1891-01

**Authors:** 


### Editorial.

### ANOTHER APPEAL.

We begin the new year with fresh hope that our efforts will secure still greater success, as a medium of communication for the thinking and working men of the profession, whose high aim is to do what they can for science and humanity. There is much to inspire them with confidence and resolution to press forward to the front ranks where honors and glory await the faithful.

But there is a strange timidity that prevents southern doctors from reporting their experience and observations.

Some of the most interesting and important cases we ever knew, occurred in country practice, and but few of them were ever published. It is surprising that so much material is wasted and lost, even now, that if reported would prove of immense value. One of the great objects of the SOUTHERN MEDICAL RECORD is to build up the Medical literature of the South. It is a fact that if gentlemen fail to write when young they will certainly not write when they grow old. Therefore we appeal to the young men again, to keep a record of their cases for publication. The rapid development of advanced ideas, should awaken a new interest in every Medical mind as to the possibilities and capabilities of the future. We will not predict, but will endeavor to keep the Medical public advised of every important truth newly discovered. We do not believe it to be the duty of a journalist to herald forth theories wrapped in mystery or beclouded with uncertainty, but to await positive results made sure by the test of time. We are in no great haste in making up our opinion, but prefer to sound the shallows and depths of every question that finds its way to us, that we may be certain of presenting the truth. Every thing should be based on facts, hasty and inconsiderate legislation is bad for the country, and theories founded upon insufficient trial or experiment are bad for science. The clearest proof is required to establish a medical fact, and still there may remain some unexplained point which may at last come to light. For instance our fathers spake of viruses which caused certain specific diseases, but they knew nothing of the factors producing these poisons and causing contagions.

The discovery of bacteria or the micro organisms has produced a new epoch in medicine; has clothed it in different and more costly apparel; has opened wide the golden gates of science, where we may enter and drink our fill of her ever bubling fountains—Young man, to the front—there is plenty of work for you. The pages of this Journal are open to you, and you are especially invited to use them.

To our contributors and subscribers we hereby tender our sincere thanks, hoping that they will continue their communications and patronage.

### LA GRIPPE.

We have been asked to give our experience with epidemic influenza or "La Grippe." By unwilling to simply trust to our own observation, we have asked a number of the leading physicians of the city for short remarks on the subject, which we print on page 49, hoping they may be of use to some of our numerous subscribers.

